# Multicenter Clinical Evaluation of the Luminex Aries Flu A/B & RSV Assay for Pediatric and Adult Respiratory Tract Specimens

**DOI:** 10.1128/JCM.00318-17

**Published:** 2017-07-25

**Authors:** Stefan Juretschko, James Mahony, Richard S. Buller, Ryhana Manji, Sherry Dunbar, Kimberly Walker, Arundhati Rao

**Affiliations:** a Northwell Health Laboratories, Pathology and Laboratory Medicine, Lake Success, New York, USA; b McMaster University, Hamilton, Ontario, Canada; c Washington University School of Medicine, Department of Pediatrics, St. Louis, Missouri, USA; d Luminex Corporation, Austin, Texas, USA; e Scott and White Medical Center, Baylor Scott and White Healthcare, Temple, Texas, USA; Boston Children's Hospital

**Keywords:** PCR, influenza, rapid tests, respiratory syncytial virus

## Abstract

Influenza A and B viruses and respiratory syncytial virus (RSV) are three common viruses implicated in seasonal respiratory tract infections and are a major cause of morbidity and mortality in adults and children worldwide. In recent years, an increasing number of commercial molecular tests have become available to diagnose respiratory viral infections. The Luminex Aries Flu A/B & RSV assay is a fully automated sample-to-answer molecular diagnostic assay for the detection of influenza A, influenza B, and RSV. The clinical performance of the Aries Flu A/B & RSV assay was prospectively evaluated in comparison to that of the Luminex xTAG respiratory viral panel (RVP) at four North American clinical institutions over a 2-year period. Of the 2,479 eligible nasopharyngeal swab specimens included in the prospective study, 2,371 gave concordant results between the assays. One hundred eight specimens generated results that were discordant with those from the xTAG RVP and were further analyzed by bidirectional sequencing. Final clinical sensitivity values of the Aries Flu A/B & RSV assay were 98.1% for influenza A virus, 98.0% for influenza B virus, and 97.7% for RSV. Final clinical specificities for all three pathogens ranged from 98.6% to 99.8%. Due to the low prevalence of influenza B, an additional 40 banked influenza B-positive specimens were tested at the participating clinical laboratories and were all accurately detected by the Aries Flu A/B & RSV assay. This study demonstrates that the Aries Flu A/B & RSV assay is a suitable method for rapid and accurate identification of these causative pathogens in respiratory infections.

## INTRODUCTION

Respiratory infections are a significant cause of morbidity, mortality, hospitalization, and health care costs worldwide (1). These infections cause acute local and systemic illnesses that range in severity and have the potential to cause severe disease, especially in the young and elderly. The vast majority (90%) of these are viral in nature (2, 3), although as many as 60% of patients are ineffectively treated with antibiotics for these viral infections (4).

The economic impact of overprescribing antibiotics is 2-fold, including the initial costly misuse of the antibiotics plus the ultimate cost of treating antibiotic-resistant infections (ARI) later on. Such occurrence of ARI has been estimated to cost more than $20,000 per infected patient (5). The clinical impact of overprescribing antibiotics includes the increased spread of viral infections, increased bacterial drug resistance, and increased cost (6). Knowing whether a respiratory infection is viral or bacterial allows for more effective patient isolation and infection control (1).

Respiratory viruses such as influenza virus and respiratory syncytial virus (RSV) are the most common cause of acute illness and physician visits in the United States (7). The frequency of respiratory viral infections is highest in children under 4 years of age. School-age children average 5 to 8 respiratory virus infections per year, and adults average 2 to 4 infections per year (8, 9). In the United States, an estimated 200,000 people are hospitalized annually due to influenza alone, with more than 36,000, typically the elderly and immunocompromised, dying from the disease each year (10).

Traditional laboratory tests, such as culture, serology, and direct immunofluorescence, have been used to identify the infectious pathogens. However, the slow results from culture and the poor reliability of rapid immunoassay-based respiratory tests present challenges to clinicians and hospitals (1). Molecular diagnostic tests such as PCR methods offer high sensitivity, speed, and cost advantages over traditional methods, such as culture or direct immunofluorescence (DFA), and have been the focus of clinical laboratories for more than a decade. Rapid and early diagnosis of the causative pathogen in respiratory illness aids in patient diagnosis, treatment management, and the avoidance of overprescribing antibiotics.

The Aries Flu A/B & RSV assay (Luminex Corporation) is a real-time PCR-based *in vitro* diagnostic test for the identification of influenza A virus, influenza B virus, and respiratory syncytial virus (RSV) in nasopharyngeal swabs (NPS) from patients with suspected respiratory tract infections. The assay is used with the Aries instrument, a clinical multiplex test system that automates nucleic acid preparation from a clinical sample, performs real-time PCR detection, and reports multiple test results from the assay. The NPS specimen is introduced into the Aries Flu A/B & RSV assay cassette by pipetting, and the cassette is placed into an Aries system module via a cassette magazine. Within the assay cassette, the specimen is lysed and the nucleic acids are extracted along with the sample processing control (SPC) present in the cassette. The extracted nucleic acids are transferred to lyophilized PCR reagents contained within the PCR tube attached to the cassette.

The assay uses target-specific fluorescently labeled primer pairs to amplify nucleic acid sequences from influenza A/B virus and RSV found in the sample, in addition to amplifying assay internal control sequences. The Aries assay chemistry is based on an expanded genetic alphabet technology, using the synthetic DNA base pair 2′-deoxy-5-methyl-isocytidine (iC) · 2′-deoxyisoguanosine (iG). The isobases (iC and iG) pair specifically with each other and not with natural nucleotides and are efficiently incorporated during PCR (11, 12). During PCR amplification, a quencher-modified iGTP is incorporated by the polymerase opposite to an iC and a fluorophore reporter attached to the PCR primer. If the target is present and is amplified, the assay fluorescence decreases with every cycle as the amplification product accumulates. The decrease in assay fluorescence is monitored in real time on the Aries instrument.

Reported herein are the results of a multicenter clinical performance study that evaluated the Aries Flu A/B & RSV assay over a 2-year period on 2,479 pediatric and adult subjects with suspected respiratory infections presenting at four different North American clinical institutions. The clinical performance of the Aries Flu A/B & RSV assay for detecting influenza A virus, influenza B virus, and RSV was determined in comparison to that of the FDA-approved xTAG respiratory viral panel (RVP).

## RESULTS

A total of 2,504 NPS specimens from subjects suspected of having respiratory tract infections were collected in the prospective study. Twenty-five specimens were excluded based on inclusion/exclusion criterion violation or protocol deviation, leaving a total of 2,479 eligible unique specimens for subsequent data analysis. Of these, 1,017 were collected from January to March 2015, while the remaining 1,462 specimens were obtained between November 2015 and February 2016.

Table 1 summarizes the general demographic information from the subjects that were included in the data analysis. Among the 2,479 specimens included in the analysis, 47.1% were from male patients (*n* = 1,168) and 52.9% (*n* = 1,311) were from female patients. Most of the specimens were collected from individuals who either were hospitalized or presented to the institutions' emergency departments (52.4% and 28.9%, respectively). Outpatients represented 18.7% of the study population. A total of 1,577 (63.6%) subjects were adults 21 years of age or older, while the remaining 902 (36.4%) subjects were pediatric patients <21 years of age.

**TABLE 1:** General demographic details of the study population

Characteristic	No. (%) at site (location):				
	1 (NY)	2 (TX)	3 (MO)	4 (ON)	All sites
Sex					
Male	375 (46.2)	134 (41.0)	200 (51.7)	459 (48.1)	1,168 (47.1)
Female	436 (53.8)	193 (59.0)	187 (48.3)	495 (51.9)	1311 (52.9)
Total	811	327	387	954	2479
Age (years)					
<1	37 (4.6)	32 (9.8)	102 (26.4)	263 (27.6)	434 (17.5)
1–5	29 (3.6)	29 (8.9)	62 (16.0)	103 (10.8)	223 (9.0)
>5–21	29 (3.6)	49 (15.0)	64 (16.5)	103 (10.8)	245 (9.9)
>21–65	316 (39.0)	131 (40.1)	124 (32.0)	274 (28.7)	845 (34.1)
>65	400 (49.3)	86 (26.3)	35 (9.0)	211 (22.1)	732 (29.5)
Total	811	327	387	954	2,479
Subject status or placement					
Outpatient	147 (18.1)	167 (51.1)	85 (22.0)	64 (6.7)	463 (18.7)
Hospitalized	484 (59.7)	111 (33.9)	158 (40.8)	546 (57.2)	1,299 (52.4)
Emergency department	180 (22.2)	48 (14.7)	144 (37.2)	344 (36.1)	716 (28.9)
Long-term care facility	0 (0.0)	1 (0.3)	0 (0.0)	0 (0.0)	1 (0.0)
Total	811	327	387	954	2479
Immune status					
Immunocompromised^*a*^	177 (21.8)	87 (26.6)	0 (0.0)	84 (8.8)	348 (14.0)
Immunocompetent	628 (77.4)	239 (73.1)	0 (0.0)	870 (91.2)	1,737 (70.1)
Not determined	6 (0.7)	1 (0.3)	387 (100.0)	0 (0.0)	394 (15.9)
Total	811	327	387	954	2,479

a Defined as patients with HIV or AIDS, transplant, oncology, or diabetes.

Clinical runs using the Aries Flu A/B & RSV assay were conducted between December and March 2016 on specimens that were either stored frozen at −80°C (*n* = 1,316 [53.1%]) or kept refrigerated at 4°C to 8°C (*n* = 1,163 [46.9%]) prior to testing. The vast majority of the frozen samples (77.3% [*n* = 1,017]) was kept stored for 3 to 12 months, while 23.6% of samples were collected 1 to 3 months before testing with the Aries Flu A/B & RSV assay. A difference in sensitivity with regard to the age of frozen specimens could not be detected. Similarly, when comparing storage conditions, no significant difference between the frozen and refrigerated sample results could be seen (data not shown).

Valid Aries Flu A/B & RSV assay results (i.e., positive or negative) were obtained on the first attempt from 2,458 specimens (2,458/2,479 [99.2%]). Invalid assay results were reported from 21 specimens (21/2,479 [0.8%]). All 21 specimens in question generated valid Aries results upon repeat testing. The positive rate of the Aries Flu A/B & RSV assay for each viral target is summarized in Table 2. Among the 2,479 specimens tested, 13.5% were positive for influenza A virus (*n* = 334) and 12.3% were positive for RSV (*n* = 306). The prevalence of influenza B virus in the study population was low (2.4% [*n* = 59]). The majority of RSV infections (*n* = 194 [63.4%]) were reported in pediatric subjects <21 years of age, while most influenza A cases (*n* = 247 [73.9%]) were from adults 21 years of age or older. Influenza B infections were evenly distributed between age groups (49.1% in pediatric cases versus 50.9% in adult cases).

**TABLE 2:** Positivity rate of Aries Flu A/B & RSV by virus and age group

Detected virus	Age (years)											
	All (*n* = 2479)		0–1 (*n* = 434)		>1–5 (*n* = 223)		>5–21 (*n* = 245)		>21–65 (*n* = 845)		>65 (*n* = 732)	
	*n*	%	*n*	%	*n*	%	*n*	%	*n*	%	*n*	%
Influenza A	334	13.5	17	3.9	30	13.5	40	16.3	151	17.9	96	13.1
Influenza B	59	2.4	4	0.9	5	2.2	20	8.2	19	2.2	11	1.5
RSV	306	12.3	133	30.6	47	21.1	14	5.7	39	4.6	73	10.0
Total	699	28.2	154	35.5	82	36.8	74	30.2	209	24.7	180	24.6

Table 3 summarizes the performance characteristics of the Aries Flu A/B & RSV assay using the xTAG respiratory viral panel (RVP) as the comparator. Clinical sensitivity (or positive percent agreement [PPA]) of the Aries Flu A/B & RSV assay was 95.8% for influenza A virus (299/312; 95% confidence interval [CI], 93.0% to 97.8%), 93.8% for influenza B virus (45/48; 95% CI, 82.8% to 98.7%), and 97.1% for RSV (270/278; 95% CI, 94.4% to 98.7%). The clinical specificity (or negative percent agreement [NPA]) of the Aries Flu A/B & RSV assay for all three respiratory pathogens ranged from 98.4% to 99.4%. Clinical sensitivity values by testing site ranged from 93.8% to 100% for influenza A virus, from 85.7% to 100% for influenza B virus, and from 95.7% to 100% for RSV. Clinical specificity values by testing site ranged from 97.4% to 99.5% for all targets. No significant differences in assay sensitivity and specificity were reported in different age groups (0 to 1, >1 to 5, >5 to 21, >21 to 65, and >65 years of age) (data not shown). Similarly, no substantial differences in sensitivity and specificity values for all three targets could be noticed between the two seasons of testing, namely, 2014 to 2015 and 2015 to 2016.

**TABLE 3:** Aries Flu A/B & RSV assay clinical performance summary in comparison to xTAG RVP assay^*a*^

Virus	Sensitivity (PPA [%])		Specificity (NPA [%])		Positive predictive value (%)		Negative predictive value (%)	
	TP/(TP + FN)	95% CI	TN/(TN + FP)	95% CI	TP/(TP + FP)	95% CI	TN/(TN + FN)	95% CI
Influenza A^*b*^	95.8 (299/312)	93.0–97.8	98.4 (2,131/2,165)	97.8–98.9	89.8 (299/333)	86.0–92.8	99.4 (2,131/2,144)	99.0–99.7
Influenza B	93.8 (45/48)	82.8–98.7	99.4 (2,417/2,431)	99.0–99.7	76.3 (45/59)	63.4–86.4	99.9 (2,417/2,420)	99.6–99.9
RSV	97.1 (270/278)	94.4–98.7	98.4 (2,165/2,201)	97.7–98.9	88.2 (270/306)	84.1–91.6	99.6 (2,165/2,173)	99.3–99.8

a FN, false negative; FP, false positive; NPA, negative percent agreement; PPA, positive percent agreement; TN, true negative; TP, true positive.

b Two specimens yielded invalid results for influenza A virus upon repeat xTAG RVP testing; both specimens were excluded from the assay performance calculations for this virus.

The positive predictive value (PPV) of the Aries Flu A/B & RSV assay was 89.8% for influenza A virus (299/399; 95% CI, 86.0% to 92.8%), 76.3% for influenza B virus (45/59; 95% CI, 63.4% to 86.4%), and 88.2% for RSV (270/306; 95% CI, 84.1% to 91.6%). The negative predictive values (NPVs) of the Aries Flu A/B & RSV assay for all three respiratory pathogens ranged from 99.4% to 99.9%. It is important to note that predictive values should not be considered intrinsic to any test, as they vary depending on the disease prevalence. Although the PPV of the Aries assay for influenza B virus was lower than those for influenza A virus and RSV, the prevalence of this virus (1.9%) was also observed to be lower in the study population than for influenza A virus (12.6%) and RSV (11.2%). This had a significant impact on PPV despite the fact that the false positivity rate of the assay for influenza B virus was only 0.6% in this evaluation (14/2,479).

Twenty-four specimens were identified as positive by the comparator method but negative by the Aries Flu A/B & RSV assay (i.e., false negative). Of these, 10 specimens (41.7%) were confirmed as negative by bidirectional sequencing analysis. These included 7 for influenza A virus, 2 for influenza B virus, and 1 for RSV. There were also 84 specimens that were identified as negative by the reference method but positive by the Aries Flu A/B & RSV assay (i.e., false positive). Of these, 39 specimens (46.2%) were confirmed as positive by sequencing analysis. These included 4 for influenza A virus, 3 for influenza B virus, and 32 for RSV.

Because the prevalence of influenza B virus was lower than those for influenza A virus and RSV in the study population, the prospective sample set was supplemented with archived specimens. A total of 40 unique preselected specimens positive for influenza B virus were included in this supplemental evaluation. The specimens were tested in a blind manner by the prospective study sites, and all were accurately detected by the Aries Flu A/B & RSV assay (100%; 95% CI, 91.2% to 100%). When the results from these banked specimens were combined with those from the prospective study, the detection rate of the Aries Flu A/B & RSV assay for influenza B virus was 96.6% (85/88; 95% CI, 90.4% to 99.3%).

## DISCUSSION

A rapid detection of viral pathogens causing a respiratory infection provides physicians a number of advantages in treating patients and managing outbreaks. In addition to confirming an infection's viral basis and avoiding unnecessary antibiotics, differential diagnosis of respiratory infections enables the isolation of hospital patients, a reduction in the length of stay for minor infections, better control of outbreaks and hospital transmission rates, and more cost-effective clinical outcomes (13, 14).

Traditional laboratory tests have limitations in the hospital setting, including a slow time-to-result, poor assay performance, and high cost (1). Without rapid test results, hospitalized patients either are not immediately isolated or are unnecessarily isolated as a precaution, potentially creating inefficiencies and increased health care costs. A poor sensitivity or specificity of an assay results in either inaccurate diagnoses or a lack of confidence in results. High assay costs make certain laboratory tests unfeasible and shift the financial burden to other aspects of clinical care, such as the decision to risk a potential outbreak or to isolate patients merely as a precaution (15).

The advent of rapid molecular diagnostic tests for respiratory viruses allows clinicians to gain diagnostic insight early enough in treatment to immediately and effectively impact clinical outcomes. However, such tests are only as effective as their proven assay performance, providing confidence and reliability for making the best possible clinical care decisions. This multicenter clinical study demonstrated robust performance of the Aries Flu A/B & RSV assay in subjects suspected of having respiratory tract infections. The primary metrics addressed by this study are the clinical sensitivity and specificity compared with those from existing molecular diagnostics for these pathogens. Other assay performance characteristics such as sample-to-answer time, ease of use, and a reduction of errors were evaluated in terms of their impact on the usage of the assay in the clinical setting.

The Aries system is a standalone sample-to-answer platform that does not require additional equipment or computing power, minimizing the system's footprint on the laboratory bench. The system is capable of running cassette-based assays, including both *in vitro* diagnostic (IVD) assays and user-defined protocols. With onboard nucleic acid extraction, internal controls, and barcode scanning, the instrument is designed to minimize laboratory errors. The Flu A/B & RSV assay requires less than 10 min of hands-on time, and results are available in approximately 2 h of run time for up to 12 samples per instrument, allowing assay results for all 3 pathogens to be reported for up to 48 patient samples per 8-hour shift.

Reliability is an essential component when choosing the right assay for any clinical setting. Unwanted repeat runs due to invalid results not only disrupt the laboratory workflow but can also lead to extended turnaround times or even cancellation of testing if insufficient sample material is available for reruns. This is especially important during seasonally driven high volumes and for decision making for triaging. The Aries Flu A/B & RSV assay demonstrated a remarkably low value of 0.8% (21/2,479) of invalid results. All of those 21 results needed to be repeated, but provided reliable results upon repeat. Similar or inferior reliability data are seen with comparable assays; GenMark Diagnostics eSensor RVP showed a 1.5% invalid rate, the Verigene RV+ Nanosphere showed 9.7%, FilmArray RP (BioFire Diagnostics) showed 3% and 1%, and Prodesse Pro Flu+ showed 5.7% (16–18). xTAG RVP (Luminex) performed with a 1.1% invalid rate in this study and 2% elsewhere (19). Even the point-of-care approved assay Alere i showed invalid rates of 4.6% and 5.1% in two studies, with the inability of reruns due to an insufficient quantity of specimen (20, 21).

Following discordant analysis and resolution, the performance of the Aries Flu A/B & RSV assay ranged from 97.7 to 98.1% sensitivity and 98.6 to 99.8% specificity for all three pathogens, which is comparable to other U.S. FDA-cleared molecular respiratory viral panels as reported in the scientific literature. For example, the study by Popowitch et al. compared four commercially available molecular tests: FilmArray RP (BioFire Diagnostics), eSensor RVP (GenMark Dx), xTAG RVP, and xTAG RVP FAST (Luminex Molecular Diagnostics) (22). Clinical sensitivities for each test evaluated varied between 86.2% to 100% for influenza A virus, 45.5% to 100% for influenza B virus, and 86.4% to 100% for RSV. Specificity values were also reported to be high for all assays, at 99.2 to 99.9% overall. Recently, Voermans et al. compared the performance of the Aries Flu A/B & RSV assay (RUO) to that of laboratory-developed reverse transcriptase PCR (RT-PCR) assays (LDA), immunochromatographic assays, and a direct fluorescent antibody. Clinical sensitivities were 93.3 to 98.6% and clinical specificities were 100% compared with those of LDA (23).

Limitations with this study include the chosen time for specimen collection that led to the inferior amount of influenza B specimens, as they had just begun to appear in each season and subsequently demanded a further collection process. Furthermore, due to the inability to definitively identify influenza A H1N1 pdm09 strains by both assays, namely, the Aries Flu A/B & RSV and the xTAG RVP, definitive conclusions about the specific performance in the identification of different influenza A strains that are predominant during the seasons of testing cannot be drawn.

In conclusion, the Aries system and the Aries Flu A/B & RSV assay provide a rapid reliable diagnostic tool for hospitals and clinical labs needing to identify viral pathogens earlier in the treatment continuum. The integrated sample-to-answer process enables clinicians to make confident decisions about patient isolation, care, and treatment that could potentially improve clinical outcomes and reduce health care costs.

## MATERIALS AND METHODS

### Institutional and ethics reviews.

The present study was conducted under institutional review board (IRB) or research ethics board (REB) approval at all participating sites.

### Prospective specimen collection, processing, and testing.

Specimens included in this clinical study included leftover nasopharyngeal swabs (NPS) prospectively collected during the 2014 to 2015 and 2015 to 2016 influenza seasons. Clinical specimens were collected from pediatric or adult patients suspected of having respiratory tract infections who presented at four geographically diverse clinical sites located in North America. Clinical sites were located in the eastern United States (Northwell Health Laboratories, Lake Success, NY), central United States (Department of Pediatrics, Washington University, St. Louis, MO), southwestern United States (Baylor Scott and White Health, College of Medicine, Temple, TX), and Canada (Department of Pathology and Molecular Medicine, St. Joseph's Healthcare, Hamilton, Ontario, Canada). Respiratory sample collection was performed with each institution's standardized NPS collection kit and in accordance with their individual procedures. All clinical specimens were submitted fresh to the institutions' clinical laboratories and were processed as per their standard procedures and as ordered by the referring physician. Specimens that were not properly collected, labeled, transported, stored, or received timely and in good condition were excluded from the study.

Upon receipt, any leftover specimen that met these study inclusion/exclusion criteria was provided in a blind manner to an individual at the site who was not directly involved in the study. Each eligible specimen was then divided into multiple aliquots. One aliquot of the specimen was kept at the clinical sites for Aries Flu A/B & RSV assay testing. Another aliquot of the specimen was shipped to a centralized testing facility (Luminex Molecular Diagnostic, Toronto, Ontario, Canada) for comparator xTAG RVP testing. Two additional specimen aliquots were generated and stored frozen at −80°C for either repeat testing or discordance analysis.

### Banked specimen collection and testing.

Banked specimens were stored frozen at −80°C over a period of 12 months prior to testing. Due to the lower prevalence of influenza B virus observed in the prospective study, the sample set was supplemented with banked (preselected) influenza B-positive specimens derived from both seasons, namely, 2014 to 2015 and 2015 to 2016. These specimens were collected at a single clinical laboratory (collection site) located in Canada (Department of Pathology and Molecular Medicine, St. Joseph's Healthcare, Hamilton, Ontario, Canada). The presence of the expected pathogen (influenza B virus) in each of the preselected specimens was confirmed by xTAG RVP testing. To minimize bias, preselected positive specimens (*n* = 40) were distributed and tested along with an equal number of unique negative specimens in a randomized double-blind fashion at the four testing sites that were involved in the prospective study. Both positive and negative results were included in the final analysis.

### Comparator testing with the xTAG RVP assay.

An aliquot of each specimen was extracted using the NucliSENS easyMAG method (bioMérieux, Inc.), and the total nucleic acid isolates were tested by the xTAG RVP assay (Luminex Molecular Diagnostics, Toronto, Ontario, Canada) at a centralized testing facility. xTAG RVP testing was performed by trained personnel in accordance with the manufacturer's instructions provided in the kit package insert and within sample stability claims.

### Aries Flu A/B & RSV assay testing.

All specimens were assessed by the Aries Flu A/B & RSV assay (Luminex Corporation, Austin, TX) by one or two trained operators at each of the clinical sites. These operators were blind to the routine clinical results to minimize potential bias. Assay runs and allowable reruns were performed in accordance with the manufacturer's instructions. Briefly, assay cassettes were removed from the pouch and capped and the foil seal was removed. Sample tubes and assay cassettes were placed in the Sample Prep tray. The samples were vortexed briefly and 200 μl from each was pipetted into the assay cassettes. The cassette caps were closed and the loaded cassettes were placed into the cassette magazine. The loaded magazine was inserted into one of two modules in the Aries instrument and the run was started. In the first phase of the prospective study (2014 to 2015 influenza season), specimens were collected and then stored frozen at −80°C prior to being tested with the Aries Flu A/B & RSV assay. Most clinical specimens collected during the second phase of the prospective study (2015 to 2016 influenza season) were tested by the Aries Flu A/B & RSV assay at the clinical sites after being kept refrigerated at 4 to 8°C for up to 96 h after collection.

### Discordant analysis with bidirectional sequencing.

Any discordant specimens where the Aries Flu A/B & RSV assay results were different from the comparator xTAG RVP assay results were assessed by bidirectional sequencing using analytically validated primers that were directed against genomic regions different from those for the Aries Flu A/B & RSV assay. Discordant specimens were subjected to bidirectional sequencing using M13 forward and reverse primers and the Sanger dideoxy sequencing method to retrieve DNA sequences. Following extraction by the bioMérieux NucliSENS easyMAG extraction method, specimens were subjected to RT-PCR using the OneStep Qiagen reverse transcriptase PCR kit (Qiagen, Hilden, Germany). Amplicons were then treated with exonuclease I and shrimp alkaline phosphatase enzymes to remove unincorporated primers and deoxynucleoside triphosphates (dNTPs) left over from the PCRs. Dye-labeled terminator cycle sequencing reactions were performed using the BigDye Terminator v3.1 cycle sequencing kit (Thermo Fisher, Waltham, MA). Any unincorporated dye was removed using the BigDye Xterminator purification kit (Thermo Fisher). Sample electrophoresis and sequencing analysis were performed on the 3730xl analyzer (Thermo Fisher) using the 3730xl data collection software (v 3.1.1) and sequencing analysis software (v 5.4). Sequences that (i) were at least 200 bases in length, (ii) had a PHRED score greater than or equal to 20 for at least 90% of the bases, and (iii) contained fewer than 5% ambiguous base calls were considered for further analysis using BLAST (NCBI). Acceptable matches to BLAST reference sequences were those with greater than 95% query coverage and identity with an expected value (E value) less than 10^−30^ compared with the reference sequence.

### Data collection and analysis.

The performance of the Aries Flu A/B & RSV assay for detecting influenza A virus, influenza B virus, and RSV was compared with that of the xTAG RVP assay. Accuracy determinations for each viral pathogen were based on the fraction of positive (or negative) results by the comparator method, which were also positive (or negative) by the Aries Flu A/B & RSV assay. An Aries Flu A/B & RSV assay result was considered to be a true-positive (TP) or true-negative (TN) result if it agreed with the comparator method result for the pathogen in question. The exact (Clopper-Pearson) method was used to calculate 95% confidence intervals. Estimates of sensitivity (or positive percent agreement [PPA]), specificity (or negative percent agreement [NPA]), the positive predictive value (PPV), and the negative predictive value (NPV) for each pathogen were calculated based on two-by-two tables (reference method result versus result from Aries Flu A/B & RSV) for the entire prospective data set.
